# Application of EEG in the Diagnosis and Classification of Migraine: A Scoping Review

**DOI:** 10.7759/cureus.64961

**Published:** 2024-07-19

**Authors:** Lakshana Raghuraman, Shiv H Joshi

**Affiliations:** 1 Medicine, Jawaharlal Nehru Medical College, Datta Meghe Institute of Higher Education and Research, Wardha, IND; 2 Community Medicine, Jawaharlal Nehru Medical College, Datta Meghe Institute of Higher Education and Research, Wardha, IND

**Keywords:** spectral power analysis, diagnosis, alpha oscillations, microstates, migraine, e.e.g

## Abstract

Migraine is a chronic debilitating disease affecting a significant number of people, more often women than men. The gold standard for diagnosis is the International Classification of Headache Disorders-3 (ICHD-3). Authors have identified multiple tight spots in the present method of diagnosis. An alternative method of diagnosis has always been coveted. Electroencephalogram (EEG) is one of the most researched of such alternatives. The visually evoked potential is the most studied; auditory evoked potentials and transcranial direct current stimulation are also being studied. Cortical hyperexcitability and habituation deficit to sensory stimuli are some of the consistent findings. Alpha oscillations are among the most frequently studied bands; spectral analysis of EEG waves has often shown more reliable and consistent results than features read off the EEG directly. EEG microstate is a novel and promising method showing characteristic identifiable features that may help diagnose Migraine patients. An alternative to the ICHD-3 criterion for diagnosing Migraines would be instrumental in promptly diagnosing the disease. EEG is one of the most explored alternatives within which enumerable features can be used to identify Migraines, of which the most promising are EEG microstates.

## Introduction and background

Migraine, a primary headache disorder with multiple subtypes, affects daily chores. According to the Global Burden of Disease Study 2010 and 2015, it was ranked the third most prevalent disorder and the third most disability-causing disorder, respectively [1]. Migraine leads to an annual loss of $3 billion due to reduced productivity [2]. Migraine is presently diagnosed according to the International Classification of Headache Disorders-3 (ICHD-beta3) criterion. Studies suggest that this criterion may need further modification to improve its accuracy and efficiency. Other modalities to diagnose Migraines include magnetic resonance imaging (MRI), transcranial Doppler and electroencephalography (EEG), magnetoencephalography (MEG), etc. Studies on EEG show novel but varied results.

Migraine affects 12% of the global population and affects women more than men. An internet-based follow-up study states that Migraine is the second most disabling condition worldwide. Approximately 2.5% of episodic Migraineurs progress into chronic Migraine [3]. Headaches of another form may eventually progress into a Migraine or vice versa. In routine practice, Migraine is diagnosed by an experienced neurologist on account of history and clinical presentation. In cases of complex manifestation of the disease, magnetic resonance imaging (MRI) and computed tomography (CT) are used to exclude other causes of headache [4]. ICHD-3 is, at present, the gold standard for the diagnosis of Migraine. ICHD-3, according to a review evaluating its evolution over the past 45 years, has been termed invaluable for diagnosis, management, and research purposes [5]. Authors have pointed out that history is unaccountably used for the diagnosis of Migraine; the criterion for the diagnosis of Migraine only includes features from headache attacks and not Migraine as a disease [6]. As considered in ICHD-3 beta alone, the episode's features may not be sufficient to diagnose and classify headache syndromes [7] as authors also highlight the significant overlap between the diagnosis of vestibular Migraine and that of brainstem aura [8] and that history is omitted even though it plays a vital role in diagnosing Migraine [6]. ICHD-3 is a symptomatic classification; it is essential to devise an etiological classification to ensure better management of this condition [6]. An automated diagnostic system would be a boon, and much research is being conducted to instrument a more accurate and efficient diagnostic system.

A Migraine is often mistaken for a sinus headache or tension-type headache. According to a study, 97% of patients with a self-diagnosis of sinus headache met the diagnostic criteria of Migraine and responded well to sumatriptan. Specific symptoms of Migraine like occipital pain, cervicalgia, facial pain, and tension in the muscles of the neck, shoulders, and upper back overlap with those of cervicogenic headache and occipital neuralgia, increasing the likeliness of a misdiagnosis [2]. Misdiagnosing similar presenting illnesses like subarachnoid hemorrhage, ischemic stroke, and cerebral artery dissection as Migraine could have irreparable consequences [2]. A long-standing disease with a substantial global impact, like Migraine, requires a more reliable method of diagnosis. One of the proposed methods for diagnosis includes the EEG, it has been explored across the distinct phases of Migraine to look for patterns that could diagnose and categorize Migraine.

## Review

Methodology

**Figure 1:**
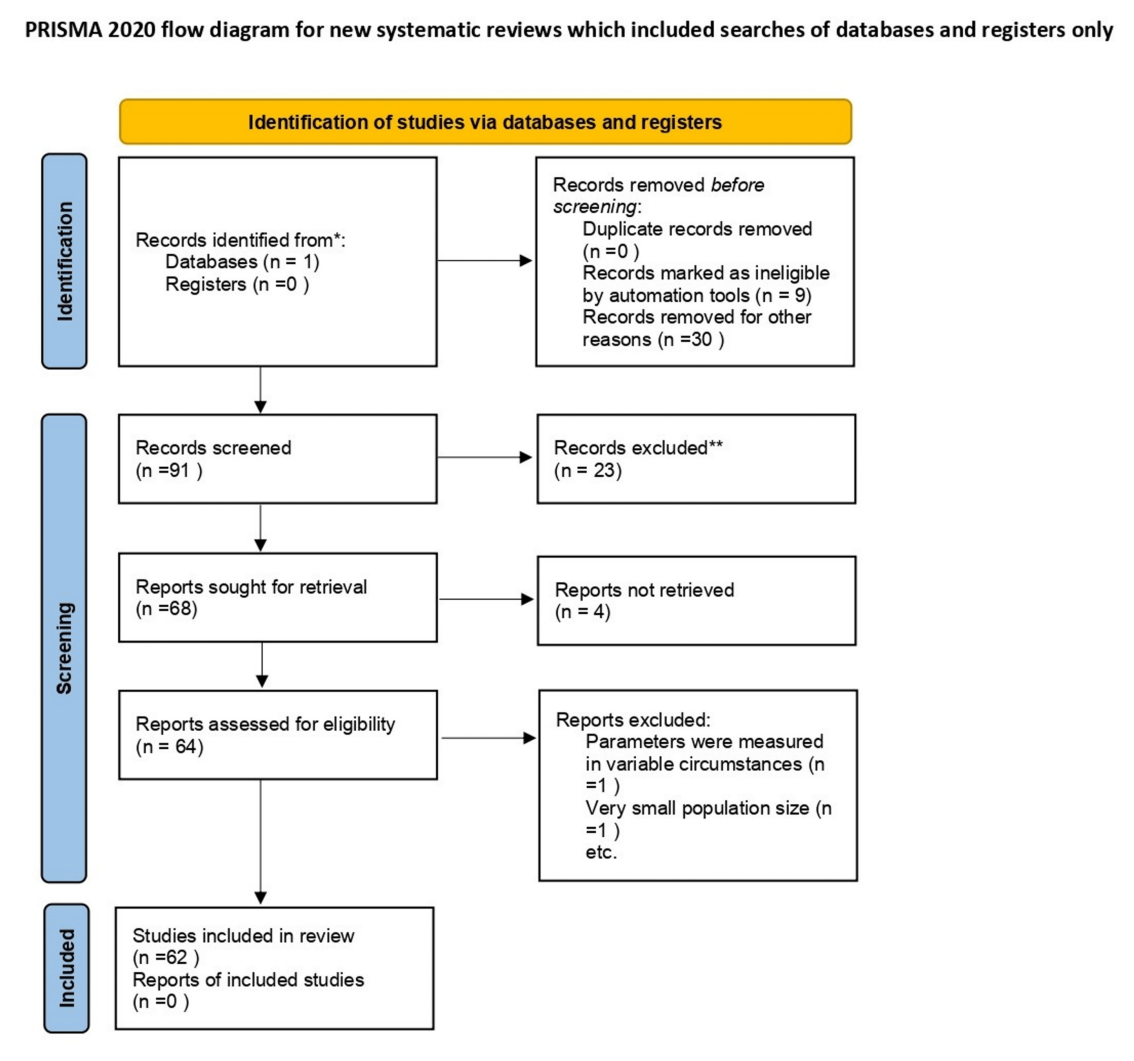
PRISMA flow diagram PRISMA: Preferred Reporting Items for Systematic Reviews and Meta-Analyses. *Reporting the number of records identified from each database or register searched (rather than the total number across all databases/registers). PubMed was the only database searched, and 91 records were identified. **No automation tools were used; all records were examined and excluded by the author.

PubMed was extensively searched for the terms "Migraine" and "EEG," excluding the words "epilepsy," "seizures," and "depression" and their associated MeSH terms. Google patents were referred to look for any previously established criterion that used EEG to diagnose or classify Migraines. All articles (systemic reviews, meta-analyses, case reports, case series, and original research) were reviewed to catch the possible use of EEG for diagnosing Migraine. Preferred Reporting Items for Systematic Reviews and Meta-Analyses (PRISMA) flow diagram of the review on EEG in the early diagnosis of Migraine is shown in Figure 1.

Components of EEG waves

EEG is usually classified according to its frequency bands. Some fundamental waveforms include delta (0.5 to 4 Hz) seen during deep sleep in frontocentral head regions; theta (4 to 7 Hz) also seen in the frontocentral head regions in early stages of sleep and drowsiness; and alpha rhythm, the characteristic pattern of the normal background rhythm of the EEG seen in the occipital region (8 to 12 Hz). Slowing down of the background rhythm indicates cerebral dysfunction. Mu alpha rhythm which is asymmetric and asynchronous is present exclusively in adolescents over the central regions. Sigma, also called sleep spindles, is seen during the N2 phase of sleep in the frontocentral areas (12 to 16 Hz). Beta, the most prominent rhythm of the EEG, has a comparatively low amplitude and is present in the frontocentral regions (13 to 30 Hz). Apart from those routinely studied (0-70 Hz), some frequencies have recently been thought to have clinical importance. This includes infra-slow oscillations and high-frequency oscillations [9].

Patterns of EEG associated with Migraine

Habituation Deficit

A phenomenon called habituation deficit (sensory modalities) is seen in electrode N75 in Migraineurs [5]. The presence of habituation deficit has been recorded in visually evoked potential at the occipital cortex of Migraineurs. Abnormalities observed in the auditory cortex include slow acoustic timing in Migraineur children [10], low otoacoustic emissions specifically in lower temporal frequencies in Migraineur women [11], and abnormal auditory brainstem responses concurrent with reduced gray matter [12-14].

Hyperexcitability and the Possible Fallacy of Hypoexcitability

Altered visual evoked potential at the occipital cortex, a common finding in Migraineurs, indicates hyperexcitability [15-17]. Decreased habituation to visually evoked gamma components during the inter-ictal period of Migraine patients is a cause for Migraine attacks. Ironically, some studies show evidence of hypoexcitability of the sensory cortex, mainly the primary visual cortex, during the pre-ictal phase of Migraine; this is contradictory to the neural hypothesis of Migraine where there is hyperexcitability of the sensory cortex. These findings are thought to be due to multiple confounding factors in these studies and the lack of grouping different subcategories of Migraine patients [18]. Table 1 summarizes the changes observed in the alpha frequency band of EEG.

**Table 1:** Changes explicitly observed in the alpha frequency region

Phase of Stimulus			Response in Migraineurs
Phase of response	Phase of stimulus		Alpha waves
Immediate responses	Period of anticipation (prior to)		Reduced alpha power (hyperexcitable as compared to normal individuals)
	Response to stimulus	High frequency	No significant differences between Migraineurs and normal individuals
		Middle frequency	
		Low frequency	
	Post-stimulation (alpha suppression)		No significant differences
Response to repeated stimulation	High frequency		Alpha suppression was more marked in Migraineurs especially in the middle frequency band
	Middle frequency (the band of maximum discomfort)		
	Low frequency		

Changes Explicitly Observed in the Alpha Frequency Region

The EEG's alpha waves have been studied extensively but less often in response to active sensory stimulus and often in resting state. The following features were observed while assessing alpha frequency oscillations over the various phases of Migraine on applying a sensory stimulation. Firstly, the period of anticipation or post cue to pre-stimulus, where the subjects were asked to fix their gaze on a screen displaying a target spot. No significant differences were observed in the alpha activity of Migraine suffers and controls at the baseline [19] but when the display portrayed the target, i.e., pre-stimulus alpha power was lower in Migraineurs [19]. Consequently, grated stimuli of different frequencies were displayed (low frequency (LF), middle frequency (MF), and high frequency (HF)). During this, the patient kept a manual count of 8 s and clicked left to move to the next stimulus or clicked right when the stimulus had undesirable effects [19]. The visual cortex of Migraineurs was at a more excitable state than controls, depicted by a significantly reduced alpha power before the onset of visual grating, i.e., the period of anticipation. No significant difference was observed in post-stimulus alpha suppression between Migraineurs and non-Migraineurs. In the second half of the experiment, alpha suppression was more remarkable in Migraine patients on prolonged visual stimulation, especially in the MF band, the band of maximum discomfort [19]. The physiology behind the increased excitability in Migraineurs can be attributed to the decreased alpha oscillation that indicates the decreased activity of inhibitory neurons in the visual cortex [20-22]. Another factor adding on to show hyperexcitability of the sensory cortex in Migraineurs is that they have a faster reaction time to stimulus than controls [23]. Some studies also suggest an inverse relationship between Individual alpha frequency and duration of chronic Migraine [18,24].

Other Observations

The low-frequency oscillations (theta and delta) are the feedback signals from the visual cortex (or other sensory areas) to the posterior regions of the thalamus. Hence, they regulate the excitability of the cortex. Studies show decreased power of these low-frequency bands in Migraine patients compared with controls, thus decreased inhibition to afferent sensory stimulus, hence the manifestation of Migraine [25-27]. Harmonized large amplitude visually evoked potential was observed in the inter-ictal phase of Migraineurs in different cortical areas as well as the occipital cortex on the application of visual stimuli. This abnormal functioning of the visual system is proposed to explain the visual aura [28,29].

A study explicitly emphasizes the relationship between total gamma power and low-frequency power spectrum. Feed-forward or afferent signals from the lateral geniculate body (LGB) create gamma frequency oscillation to the primary visual cortex. The frequency of oscillation in the primary visual cortex is proportional to the efficiency of processing of the stimulus by the thalamocortical network and with the conversion of stimulus into coherent perception [30-34]. During Migraine attacks and chronicity, the thalamocortical network is more efficient than during headache-free intervals, as indicated by the increased gamma oscillation induced by visual stimulation [30,35-38].

During a Migraine attack, there is decreased alpha oscillation, high alpha power (induced by a sensory stimulus), and increased gamma oscillations (in response to pain). On applying a sensory stimulus, usually, a photic stimulus, when the stimulus is perceived, but attention has not yet been paid to it, the lateral geniculate body (LGB) of the posterior thalamus is activated and further sends feed-forward signals to the primary visual cortex, recorded as increased gamma power. The primary visual cortex consequently sends out low-frequency alpha, beta, and theta feedback signals that regulate the feed-forward signals by a process called gating. Migraine patients deviate from the ideal gating that occurs and prevents over excitation of the LGB followed by the primary visual cortex, the LGB and again and the trigeminal afferents that terminate at the posterior areas of the thalamus in proximity to the LGB. The dysregulation of this circuit is a postulated cause of the perceived headache.

Pathophysiological Basis of Hyperexcitability and Habituation Deficit

Connectivity alterations in multiple cortical regions are observed in Migraine's pre-ictal and ictal phases [39]. Common connectivity abnormalities observed are between areas associated with pain and between sensory areas [40]. The strength of these abnormal connectivity is proportional to the severity of the headache [41]. Considering the sensory hyperexcitability, it is postulated that connectivity alterations may be more evident on sensory stimulation [23].

**Figure 2:**
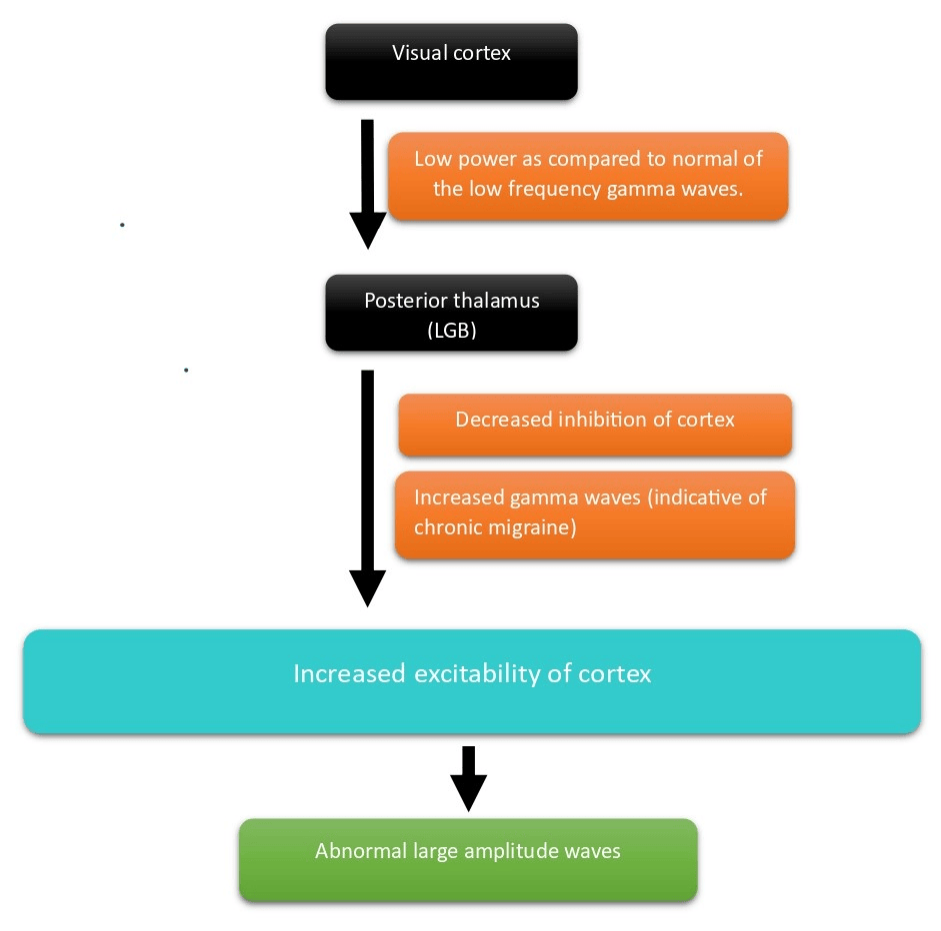
Pathophysiological basis of Migraine Authors own creations. LGB: lateral geniculate body.

In Migraineurs, there is an apparent reduced thalamic nucleus; as a result, there is decreased neural synchrony across the brain, majorly in low-frequency oscillations (theta). Reduction in connectivity in the theta band is a consistent finding and has been successfully used to classify a group into Migraineurs and non-Migraineurs [42]. Coherence is a measure of synchronization used to measure cortical connectivity by measuring the distance and frequency. It could show connectivity abnormalities in Migraine patients, especially on applying auditory or visual stimulus. Coherence is measured at various positions and interelectrode distances to analyze the connectivity of the cortex at rest and during the application of sensory stimuli. Significantly lower spatial coherence was observed more evidently in the alpha frequency band predominantly in the frontal clusters in both inter- and intra-hemispheres for mainly long inter-electrode distance for visual and auditory stimuli of both 4 Hz and 6 Hz each and at resting state and during both active Migraine attack and inter-ictal period [23]. Lower spatial coherence indicates de-synchronization which suggests greater functional activity which is consistent with the cortex being hyperexcitable [43-45]. These measures can be refined and used as a diagnostic criterion for Migraine. A deep understanding of the different connectivity changes running through the inter-ictal period flowing into the various phases of Migraine could help us develop treatment modalities and predictors of a Migraine attack or even methods to prevent attacks [46]. Figure 2 highlights the pathophysiological basis of Migraine.

It is also imperative to mention that there is a less habituation deficit than is usually reported and interpreted as repetitive stimulation by undesirable visual stimulus leads to an alpha hypersynchronization that results in a transient cortical deactivation which shows that the feed-forward system does function. A speculation responsible for the Migraine episodes would be the override of hyperexcitability and the fading of the protective gating mechanism due to the hyperexcitability [47].

Assessment of EEG power spectral density in Migraine

A study comparing EEGs of Migraine patients and healthy controls studied the relative power, median frequency, individual alpha frequency, and spectral entropy as complementary measures that must assess spectral content [48]. Consequently, the prevalence and progression of Migraine can be indicated by analyzing a statistically significant characteristic feature present over the B2 band of the alpha frequency range [18]. The author ascertains that the strength of the estimated spectral findings is inversely proportional to the duration of onset of Migraine, which explains why the results of this particular study show a strong association of relative power of the characteristic feature in the B2 band but its lack of association with chronic Migraine [18]. At rest, Migraine patients' EEGs showed a reduction in the relative power of beta 1 (11.6 to 12.8 Hz), a sub-band belonging to the high alpha range [18]. Healthy individuals and Migraine patients can be differentiated using the frequency band B1 (11.2, 14.9 Hz) of the alpha frequency waves [18]. And chronic Migraine patients and episodic Migraine can be differentiated by the presence of a characteristic B2 (26.1, 28.3 Hz) band in episodic Migraine patients [18]. Spectral power analysis can predict Migraine attacks along with portable EEG detection [49].

Microstates and Migraine

EEG microstates, also referred to as global patterns of the spatial configuration of electrical potentials that dynamically evolve in an organized manner, is a multichannel sub-second time scale that records the spatiotemporal dynamics of several components of the resting state brain [50-52]. A microstate is a 60-120 s period where a single spatial configuration is dominant or quasi-stable. It has broadly been classified into four broad categories A, B, C, and D, which accommodate 65%-84% of the total topographical variance [51]. Table 2 summarizes differences observed in the microstates of EEGs of Migraine patients in terms of mean duration, occurrence, and time coverage.

**Table 2:** Microstates and Migraine Authors own creation.

Phase of Microstate	Component Analyzed	Migraine Patients	Controls
Phase B and D	Occurrence	Occur more frequently	Occur less frequently
	Time coverage		
Phase C	Occurrence	Occur more frequently	Occur less frequently
	Time coverage		

EEG microstates parameters have shown potential utility in detecting neuropsychiatric illnesses duration, occurrence, time coverage, and syntax (likeliness to make a transition) [50,51]. A study comparing EEG microstates in Migraine without aura patients laid the following observations; mean duration of microstate class C was more remarkable in controls than in Migraine without aura patients. Frequency of occurrence and time coverage of microstate classes B and D were significantly more in Migraine without aura than in healthy controls (HC). In contrast, microstate class C is less in Migraine without aura patients than in HC [51,53-55]. Microstate class C, representing the salience network (dorsal anterior cingulate cortex and anterior insula) [56], showed a decreased time coverage, occurrence, and mean duration. These results second previous functional magnetic resonance imaging (fMRI) studies of Migraine patients [57]. The salience network filters sensory stimuli and passes them over to the central-executive network that plays a role in inhibitory control [58,59]. The aberrant connectivity of the salience network explains the decreased inhibition to sensory stimuli and the hypervigilance to salient events. Similar to microstate B, microstate D representing the dorsal areas of the frontal and parietal cortex showed both decreased time coverage and frequency of occurrence at the baseline in migraine without aura (MwoA) patients. This seconds other brain imaging studies proving hyperexcitability/exaggerated reflexive responsivity to both attended and unattended sensory stimuli [60-62].

Syntax

Decreased connectivity from the salient network to sensory areas consistent with the hyperexcitability of these areas in Migraine without aura patients is represented by the reduced number of transitions from microstate class C to classes B and A. Increased shifts were observed from microstates class D to B, consistent with increased connectivity between dorsal areas of the frontal and parietal cortex and the visual network.

Potential diagnostic use of EEG in Migraine

Gaining conscience that it is imperative to look for an alternative method for diagnosing Migraine, preferably an automated diagnostic method, EEG is one of the most promising and studied options. Other options include using transcranial Doppler along with EEG and fMRI. It is established that Migraine triggers are responsible for deviation from baseline electrical activity. There are around 15,000 non-patented studies describing various criteria for diagnosing Migraine using EEG. This area has been of great interest in the last few years. Dr. S. Batuhan Akben has proposed a very efficient method for the automatic diagnosis of Migraine; he used the (auto regressive) AR-Burg, which has popularly been used by other research for EEG signal processing. Power spectral density was obtained from four primary channels. The T3 channel (which lies over the temporal artery showed a classification accuracy of 88.7% and a kappa value of 0.77. The F7 channel (located over the frontal branch of the temporal artery was the second best accurate, with an accuracy of 85% and a kappa value of 0.70 followed by the O1 and O2 channels located over the occipital cortex. Other promising techniques used to process EEG include tunable Q factor wavelet transforming ensemble and non-linear feature extraction technique. Table 3 summarizes the findings of included articles in brief.

**Table 3:** Reference summary ICHD: International Classification of Headache Disorders, fMRI: functional magnetic resonance imaging, MEG: magnetoencephalography, GLM: general linear model, BOLD: blood oxygen level-dependent response, MWoA: migraine without aura, MwA: migraine with aura, EPOCHs: a particular time frame selected from a continuous EEG which has been divided into multiple time frames/epochs.

Sr.No.	Year	Author	Findings
1.	2017	Ambrosini et al. [2]	Analysis of EEG of visually evoked potentials with an eye to observe habituation deficit showed no deficit in healthy controls and Migraineurs during an attack, but inter-ictal habituation deficit was observed in Migraineurs, the deficit was followed up within a week, and the findings were consistent.
2.	2016	Ashina et al. [3]	The current gold standard for diagnosis of Migraine is the ICHD-beta 3; it diagnoses Migraine on the basis of various features of an attack. Over the years, Migraine is found to be associated with specific genes. This article describes and emphasizes on the need to explore the biomarkers as a tool to diagnose and classify Migraine.
3.	2009	Bjørk et al. [4]	The alpha band showed similar width, peak power, and frequency in controls and in the inter-ictal phase in Migraineurs. Inter-ictally, the alpha peak frequency is reduced and variability is increased.
4.	2015	Brighina et al. [5]	Fusion illusion was tested in Migraineurs and controls for single light and double beep stimulus. Fusion illusion was consistently reduced ictally in Migraine patients.
5.	2010	Britz et al. [6]	fMRI has an image gap pf 10 s while that if EEG microstates has 100 ms, on comparison of fMRI and EEG interpretations in four microstates, EEG microstate correlates of respective fMRI were observed to fluctuate more frequently than fMRI.
6.	2005	Brookes et al. [7]	This study compares the MEG analyzed using the GLM beam former technique analyzing both phase locked and non-phase locked neuromagnetic effects and fMRI. It focuses on the BOLD and transient responses. It highlights the need to conduct further studies comparing the same in stationary field and changes in oscillatory field to get a better understanding of the relationship between neuronal activation and hemodynamic response.
7.	2014	Cai et al. [8]	This study is a meta-analysis of 70 cortical inhibitory control studies. They aimed to differentiate between the inhibitory roles of the right inferior frontal cortex and the right anterior insula. The right anterior insula was found to be associated with behavioral analysis, while the right inferior frontal cortex is instrumental in exerting inhibitory control.
8.	2021	Chamanzar et al. [9]	The abnormal patterns of EEG coherence in inter-ictal Migraineurs during visual and auditory stimuli, as well as at rest (eyes open), may be associated with the cortical hyper-responsivity that is characteristic of abnormal sensory processing in Migraineurs.
9.	2019	Chong et al. [10]	Results of this review show that headache disorders are associated with atypical functional connectivity of regions associated with pain processing.
10.	1996	Chronicle et al. [11]	An effort to establish the association between the photophobia and hypersensitivity of visual pathway as a trigger of Migraine.
11.	2016	Coppola et al. [12]	Cognitive impairment during the ictal phase is attributed to the abnormal connectivity between the thalamus and attentional cerebral networks.
12.	2018	Clayton et al. [13]	Alpha oscillations exhibit at least five distinct characters (inhibitor, perceiver, predictor, communicator, and stabilizer) that may be altered in case of Migraine patients.
13.	2013	Coppola et al. [14]	Early sensitization to response is attributed to abnormal connections across the thalamus and cortex.
14.	2017	de Tommaso et al. [15]	The strength of connection marked by the amount of information exchange in the parietal-occipital regions indicates the altered excitability of visual cortex. This is one of the mechanisms thought to be responsible for visual aura.
15.	1989	Fong et al., [16]	Dysfunction of GABAergic inhibitory mechanisms can be postulated to cause hyperresponsiveness of visual cortex in Migraine patients.
16.	2020	Frid et al. [17]	Analysis of functional connectivity metric of EEG data at the time of rest can be used as a single biomarker to differentiate between MWA and MWoA.
17.	2005	Fries [18]	A hypothesis that neuronal communication is mechanistically subserved by neuronal coherence.
18.	1994	Genco et al. [19]	Migraine with aura patients have significant delta rhythm percentage power as compared to Migraine without aura patients.
19.	2020	Gomez-Pilar et al. [20]	Spectral characteristics in different frequency bands can be used to differentiate between different Migraine subgroups.
20.	2001	Hadjikhani et al. [21]	Electrophysiological finding of cortical spreading depression is stipulated to be responsible for auras.
21.	2018	Haigh et al. [22]	Monotonical cortical excitation is observed with color alterations.
22.	2019	Han et al. [23]	Positive correlations were established between executive control networks response time and frequency or duration of Migraine attacks.
23.	2016	Hodkinson et al. [24]	Perceptions of multiple sensory modalities are disturbed. Strengthening this finding, primary sensory areas were found to maintain local functional connectivity but expressed impaired long range connections to higher-order association areas.
24.	2016	Joffily et al. [25]	The magnitude of transiently evoked otoacoustic emission was lower in Migraineurs and was statistically significant in the frequency range 1-1.5 KHz.
25.	2000	Judit et al. [26]	Deficient habituation of pattern reversal visual evoked potentials is observed in Migraineurs.
26.	2015	Khanna et al. [27]	EEG microstates is an evolving tool showing promising results for the diagnosis and classification of Migraine.
27.	2006	Kruit et al. [28]	Infratentorial hyperintensities (pontine) were observed in Migraineurs.
28.	1987	Lehmann et al. [29]	Adaptive segmentation of map series of EEGs into spatially stationary EPOCHs can assist in the diagnosis and classification of Migraine.
29.	2020	Lipton et al. [30]	Latent class analysis can be used to identify and differentiate Migraine subgroups.
30.	2020	Lisicki et al. [31]	Abnormalities in feedback and feed-forward visual signaling in Migraine patients differ with the presence of headache.
31.	2006	Loder and Rizzoli [32]	The use of biomarkers as a predictor, diagnostic, prognostic marker of Migraine, and its limitations.
32.	2015	Manzoni and Torelli [33]	Highlights of the need for more systematization in the classification of chronic Migraine.
33.	2018	Marciszewski et al. [34]	Migraine patients may have brainstem anatomy changes lay ground to the activation of ascending trigeminal pathway during Migraine attacks.
34.	2020	Martins et al. [35]	Wearable EEGs can be used as a preattack predictor and thus aid subsequent early intervention.
35.	2020	Masson et al. [36]	Auditory networks are also found to have altered patterns of EEG as compared to controls.
36.	2019	Mehner et al. [37]	Migraineurs are observed to show increased occipital responsiveness to visual stimuli as compared to controls.
37.	2018	Michel and Koenig [38]	Microstates (quasi-stable states) which while read paying attention to the intricate milliseconds show certain artifacts that can be used to differentiate between Migraineurs and controls, this is a novel tool that can be extrapolated for the diagnosis of Migraine.
38.	2016	Mickleborough et al. [39]	fMRI shows anatomical differences in the attentional networks Migraineurs and non-Migraineurs.
39.	2011	Mickleborough et al. [40]	Describes various behavioral manifestations of the hyperexcitable visual cortex in Migraineurs as compared to controls.
40.	2011	Mickleborough et al. [41]	Activities and controls of the visual cortex are altered in Migraineurs. This article elaborates on the alterations in control of the visual cortex.
41.	2018	Mykland et al. [43]	Synchronization is altered following the beta-phase in Migraineurs.
42.	2016	Niddam et al., [45]	Neocortical and other connectivity to the visual cortex is altered such that excitability of the cortex is altered in Migraineurs.
43.	2014	Olesen [46]	Conveys the need for certain pitfalls in the ICHD-beta 3 need to be assessed and improved.
44.	2012	Olsen et al. [47]	There are six layers to the cortex and the connection of the outermost layer is the most instrumental in control of excitability.
45.	2016	Putcha et al. [48]	Salience can predict levels of cognition in aging and Parkinsons.
46.	2008	Romei et al. [49]	Alterations are observed in the posterior alpha band of EEG which reflects altered excitability of the visual cortex.
47.	2004	Sable et al. [50]	There is a latent reduction of auditory N1 amplitudes because of sound repetition.
48.	2001	Salinas and Sejnowski [51]	Synchronous oscillations are postulated to be associated with the flow of information, attention, etc.
49.	1995	Schoenen et al. [52]	Migraine is found to be associated with defective central information processing.
50.	2007	Siegel et al. [53]	MEG can be used to assess the intensity of signals. Frequency at the visual motion pathway can help encode the intensity of visual motion signals.
51.	1995	Singer and Gray [54]	The combinatorial problem solved to have resolved the puzzle of visual pathway.
52.	2009	Siniatchkin et al. [55]	Pre-Ictal changes have been observed on EEG in Migraine patients.
53.	2019	Skorobogatykh et al. [56]	fMRI can help In improved understanding in the pathophysiology of Migraine.
54.	1999	Bertrand and Tallon-Baudry [57]	Oscillatory gamma activity of the visual cortex has characteristic alterations in Migraineurs.
55.	2018	Tinsley and Rothrock [58]	Highlights of the fallbacks of the diagnostic criteria of Migraine and areas that may need attention.
56.	2014	van Kerkoerle et al. [59]	Postulates that alpha and gamma oscillations describe feed-forward and feed-backward processing, respectively, in monkey visual cortex.
57.	2012	Xue et al. [60]	fMRI at resting state can identify Migraine without aura patients.
58.	2005	Yi et al. [61]	Reasons and statistics of cervicogenic headache being misdiagnosed as Migraine.
59.	2010	Zumer et al. [62]	fMRI observations during convulsions.

## Conclusions

A study of EEG at rest, under visual stimulation and auditory stimulation rather than a single modality alone, would make our understanding of the association of EEG patterns with Migraines more insightful. The inconsistency of EEG findings in numerous studies is due to differences in data processing and the vast phases, stages, and differences in the manifestation of Migraines. Thus, a more efficient differentiating criterion could be found if the EEGs of different Migraine subgroups are studied separately. The observations seen in EEG microstates are promising. However, they have only been explicitly studied in patients of Migraine without aura and have not been studied in patients with other Migraine syndromes and hence need to be further explored.
